# An Open Question: Is It Rational to Inhibit the mTor-Dependent Pathway as COVID-19 Therapy?

**DOI:** 10.3389/fphar.2020.00856

**Published:** 2020-05-29

**Authors:** Giuseppe Terrazzano, Valentina Rubino, Anna Teresa Palatucci, Angela Giovazzino, Flavia Carriero, Giuseppina Ruggiero

**Affiliations:** ^1^Department of Translational Medical Sciences, University of Naples Federico II, Naples, Italy; ^2^Department of Science, University of Basilicata, Potenza, Italy

**Keywords:** COVID-19, hyper-activation, immune-regulation, mTOR, Everolimus

## Introduction

In December 2019, a novel coronavirus infection appeared in China (Wuhan City and Hubei Province), causing the first cases of abnormal severe pneumonia. Since then, the SARS-Cov2 infection has become pandemic and the correlated coronavirus disease (COVID-19) has been showing a plethora of pathophysiological manifestations that do not exclusively reduce COVID-19 to the occurrence of severe acute respiratory distress (Cevik et al., 2020; Chen et al., 2020; Harapan et al., 2020; Ryu and Chun, 2020; Sen et al., 2020).

Although the immunological responses against SARS-Cov2 remain poorly defined (Chen et al., 2020; Li et al., 2020; Nikolich-Zugich et al., 2020; Qin et al., 2020), it is of note that the critical phase of COVID-19 currently appears, at least in some critical pathophysiological aspects, as a sort of autoimmune disease or as immune response hypersensitivity. Consequently, many authors have proposed various therapeutic approaches based on the modulation/inhibition of abnormal immune response in COVID-19 (Askanase et al., 2020; Chen et al., 2020; Geng et al., 2020; Li et al., 2020; Nikolich-Zugich et al., 2020; Piva et al., 2020; Qin et al., 2020; Radbel et al., 2020; Whyte et al., 2020; Ye et al., 2020; Yuki et al., 2020; Zhao, 2020).

Recently, the effects of Tocilizumab administration has seemed to indicate that inhibition of the Interleukin (IL)-6 receptor (IL-6R) may result in the recovery of critical COVID-19 patients (Aziz et al., 2020; Zhang et al., 2020) in the advanced post-alveolitic phase, when extensive pulmonary fibrosis is accompanied by a diffuse interstitial inflammation apparently sustained by a described exacerbated cytokine storm (Askanase et al., 2020; Chen et al., 2020; Geng et al., 2020; Li et al., 2020; Nikolich-Zugich et al., 2020; Piva et al., 2020; Qin et al., 2020; Radbel et al., 2020; Whyte et al., 2020; Ye et al., 2020; Yuki et al., 2020; Zhao, 2020). Such evidence highlights the critical relevance of controlling the IL-6/IL-6R pro-inflammatory pathway in the pathophysiology of COVID-19 in order to mitigate the adverse immune response that is a determinant of the most serious and undesirable phase of SARS-Cov2 infection (Whyte et al., 2020; Yuki et al., 2020; Zhao, 2020). In particular, hyper-reactivity was described as a major feature of the critical phase of COVID-19, broadly due to the hyperacute inflammatory context that leads to pulmonary interstitial disease and severe acute respiratory distress (Askanase et al., 2020; Chen et al., 2020; Geng et al., 2020; Li et al., 2020; Nikolich-Zugich et al., 2020; Piva et al., 2020; Qin et al., 2020; Radbel et al., 2020; Whyte et al., 2020; Ye et al., 2020; Yuki et al., 2020; Zhao, 2020).

## The Observed Hyperactivation of the Immune Response in COVID-19 and the Hypothesis of an Everolimus Approach to Inhibit the mTor-Dependent Pathway

Taken as a whole, several datasets produced so far on COVID-19 suggest a central role for immune response dysregulation in the pathophysiological features occurring in the severe forms of this infectious disease (Askanase et al., 2020; Chen et al., 2020; Geng et al., 2020; Li et al., 2020; Nikolich-Zugich et al., 2020; Piva et al., 2020; Qin et al., 2020; Radbel et al., 2020; Whyte et al., 2020; Ye et al., 2020; Yuki et al., 2020; Zhao, 2020). Therefore, it appears legitimate to consider the use of therapies aimed at controlling/inhibiting the immune response by using drugs acting on immune homeostasis (Askanase et al., 2020; Chen et al., 2020; Geng et al., 2020; Li et al., 2020; Nikolich-Zugich et al., 2020; Piva et al., 2020; Qin et al., 2020; Radbel et al., 2020; Whyte et al., 2020; Ye et al., 2020; Yuki et al., 2020; Zhao, 2020).

In the context of immune response regulation, of great relevance is the role of regulatory T cells (Tregs), which are a subpopulation of CD4^+^ T lymphocytes involved in the control of immunological self-tolerance and in the maintenance of immune homeostasis (Sakaguchi, 2005; Strauss et al., 2007; Procaccini et al., 2010). In addition, the intracellular mammalian Target Of Rapamycin (mTOR) molecule plays a key role in immune-regulation and immune-tolerance control pathways (Wullschleger et al., 2006; Dowling et al., 2010; Fasolo and Sessa, 2014; Chen et al., 2019).

mTor is the main intracellular nutrient sensor and, a serine-threonine kinase, its activity acts to regulate the cell cycle and growth by “sensing” the extracellular energy state given by amino acids, glucose, growth factors, and hormones (Dunlop and Tee, 2009; Laplante and Sabatini, 2009; Dowling et al., 2010; Galgani et al, 2010). It favors cellular metabolism and growth when conditions are favorable or catabolic processes when conditions are unfavorable. mTOR is present in two different multi-protein complexes. 1) mTOR Complex 1 (mTORC1) (Dunlop and Tee, 2009; Laplante and Sabatini, 2009; Dowling et al., 2010), formed by the association of mTOR with Raptor, GβL, PRAS40, and Deptor. Briefly, a high level of ATP, growth factors, and hormones activate mTORC1. Conversely, low ATP levels and the absence of growth factors inhibit mTORC1. Activated mTORC1, through target phosphorylation (such as p70 S6 kinase), induces metabolic effects such as mRNA translation, ribosome biogenesis, protein synthesis, mitochondrial metabolism, and adipogenesis. 2) mTOR Complex 2 (mTORC2) (Dunlop and Tee, 2009; Laplante and Sabatini, 2009; Dowling et al., 2010), composed of mTOR, Rictor, GβL, Sin1, PRR5/Protor-1, and Deptor. mTORC2 promotes cell survival (via Akt), cytoskeleton (via PKC), ion transport, and cell growth (via SGK1).

Aberrant signaling of mTOR is involved in many pathological states, such as cancer, cardiovascular disease, inflammation, autoimmunity, and metabolic disorders (Dowling et al., 2010; Procaccini et al., 2010; Fasolo and Sessa, 2014; Chen et al., 2019).

**Figure 1:**
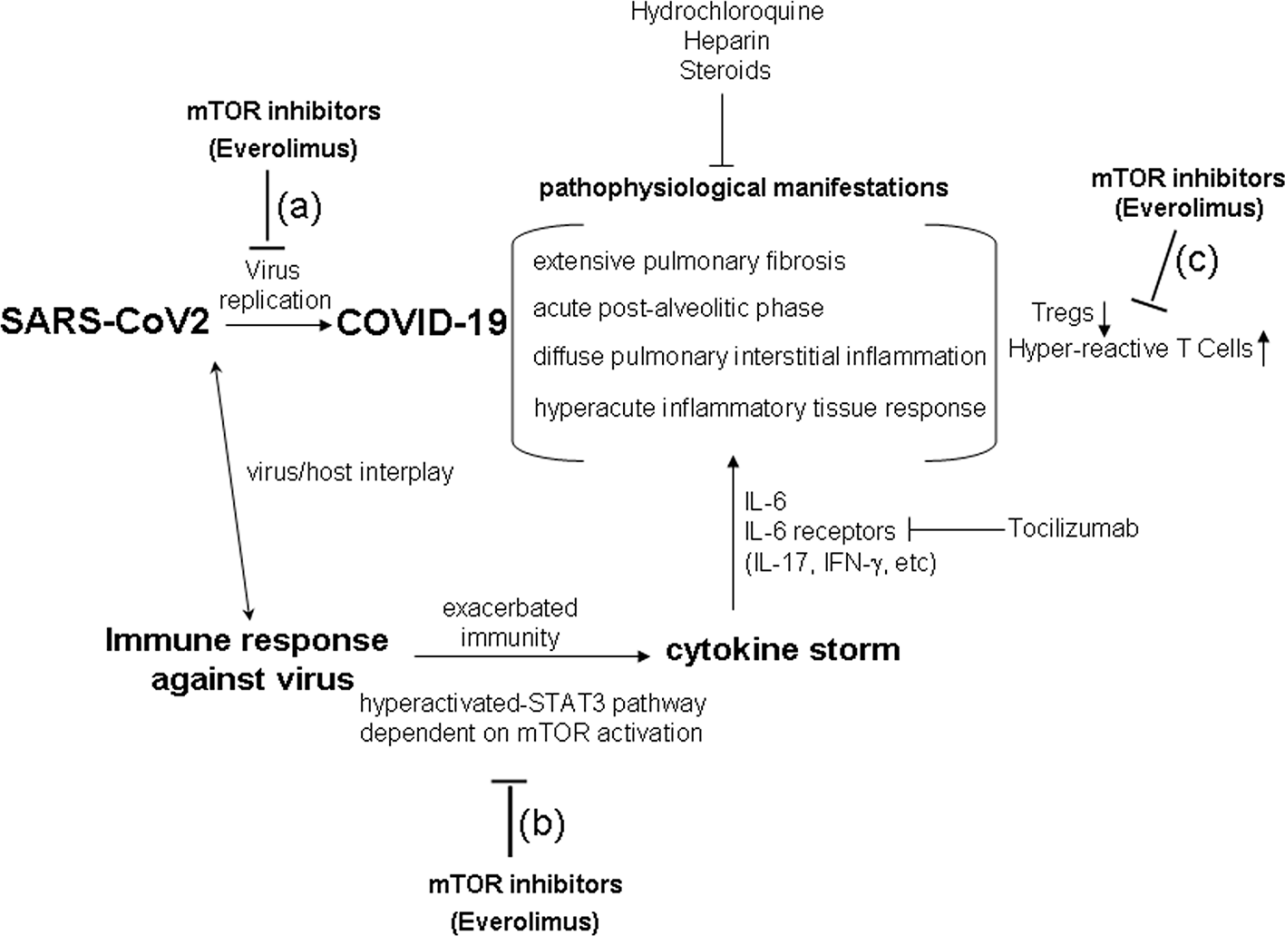
The hypothesis of a therapy using mTOR inhibitors (Everolimus) in COVID-19. The critical phases of COVID-19 show pathophysiological aspects resembling a sort of immune response hypersensitivity, mediated by exacerbated immune mechanism and cytokine storm. mTOR inhibition (i.e., by Everolimus) could act in reducing SARS-CoV2 replication **(A)**, in inhibiting the cytokine storm dependent on the hyper-activated-STAT3 pathway **(B)**, in contrasting Treg down-regulation, and in reducing conventional hyper-reactive T cells **(C)** in COVID-19. Current step-use of Tocilizumab, Hydrochloroquine, Heparin, and Steroids is reported in the figure.

Rapamycin is the prototypic mTOR-inhibitor drug (Albert et al., 2010). Several analogs of Rapamycin have been synthetized for effective therapeutic use (Albert et al., 2010). In this context, Everolimus is a promising second-generation rapamycin derivative in terms of adverse effect management (Kaplan et al., 2014). Since it is a specific inhibitor of mTORC1, the drug down-modulates mRNA translation, ribosome biogenesis, protein synthesis, mitochondrial metabolism, and adipogenesis (Dunlop and Tee, 2009; Laplante and Sabatini, 2009; Dowling et al., 2010). In this regard, mTORC1 inhibition by Everolimus is effectively used in several transplantation therapies and in a broad range of disease therapies or anti-tumoral approaches (Hernández et al., 2011; Fasolo and Sessa, 2014). Classic mTOR inhibition by Rapamycin exerts opposite effects on conventional T lymphocytes and on Tregs, and the differential impact is likely dependent on the intracellular state in the two cellular subpopulations (Strauss et al., 2007; Procaccini et al., 2010). Briefly, cell growth of conventional T lymphocytes is inhibited by Rapamycin, while such a drug expands Tregs proliferation (Strauss et al., 2007; Procaccini et al., 2010). Therefore, mTOR inhibition by drugs – and, specifically, the Everolimus-mediated mTORC1 inhibition - may also assert this differential effect in COVID-19: a reduction in proliferation of conventional T lymphocytes, which could mitigate the cytokine storm, and preserved Treg growth and activity, which could reduce the hyper-reactivity in the critical phase of the disease (**Figure 1**).

The inhibition of mTOR may prevent the immune hyperactivation of the signal *via* the STAT3 pathway that, in turn, has been described to increase the expression of receptors for IL-6 and IL-6 production itself (Wullschleger et al., 2006; Yu et al., 2007; Dowling et al., 2010; Fasolo and Sessa, 2014; Chen et al., 2019). The inhibition of mToR by Everolimus also results in a complex favorable modulation of cancer and of immune response interplay (Hernández et al., 2011; Fasolo and Sessa, 2014; Sabbatini et al., 2015; Johnson et al., 2018).

Our research group observed (Sabbatini et al., 2015) that, in kidney transplant patients undergoing conversion from the calcineurin inhibitor Ciclosporin to Everolimus, the obtained balanced mTOR inhibiting effect was able to guarantee more controlled and specific immunosuppression than calcineurin inhibitors, for example by maintaining high and qualitatively effective levels of Tregs, inhibiting the secretion of pro-inflammatory IL-17 and IFN-γ cytokines, and reducing the hyper-activation of CD8 T cells in kidney post-transplantation. Such aspects could be of some relevance also in avoiding the occurrence of pulmonary fibrosis in COVID-19 (**Figure 1**), which could be due to the cytokine storm and immune response hyper-activation (Askanase et al., 2020; Chen et al., 2020; Geng et al., 2020; Li et al., 2020; Nikolich-Zugich et al., 2020; Piva et al., 2020; Qin et al., 2020; Radbel et al., 2020; Whyte et al., 2020; Ye et al., 2020; Yuki et al., 2020; Zhao, 2020).

Moreover, Everolimus has surprisingly been associated with the reduction of viral replication of CMV, BKV, and HCV post-transplantation and in cancer patients (Garofalo et al., 2019; Nanmoku et al., 2019; Tan et al., 2019), although the specific drug mechanism has never been definitively clarified. In this regard, the combination of antiviral drugs like leflunomide and fluoroquinolones/Everolimus should favor BKV viremia clearance (Garofalo et al., 2019), and the conversion from conventional immunosuppressant drugs to Everolimus appears to induce the remission of EBV-related lymphoproliferative disorder in kidney transplantation patients (Nanmoku et al., 2019). Moreover, Everolimus has been described to effectively inhibit *in vitro* CMV replication in infected cells (Tan et al., 2019).

## Discussion

The question to be answered is whether a therapy that uses Everolimus in COVID-19 could reduce the pathophysiological hyperactivation of the immune response in the lung and other organs described as extensively degenerated by inflammation upon infection with this coronavirus (Chen et al., 2020; Li et al., 2020; Nikolich-Zugich et al., 2020; Piva et al., 2020; Qin et al., 2020; Radbel et al., 2020; Whyte et al., 2020; Ye et al., 2020; Yuki et al., 2020; Zhao, 2020).

It is certainly a gamble to administer a potentially immunosuppressive drug in a viral infection, and therefore Everolimus should probably be used at doses close to those used in anti-tumor therapy to avoid adverse effects dependent on the immune-depression emerging at higher doses. As referred to in the previous paragraph, Everolimus may inhibit conventional T lymphocytes and may maintain Treg functions to reduce hyper-reactivity in COVID-19 (**Figure 1**). However, Everolimus could be administered together with current therapeutic approaches, particularly in the critical phase of SARS-Cov2 infection (Askanase et al., 2020; Chen et al., 2020; Geng et al., 2020; Li et al., 2020; Nikolich-Zugich et al., 2020; Piva et al., 2020; Qin et al., 2020; Radbel et al., 2020; Whyte et al., 2020; Ye et al., 2020; Yuki et al., 2020; Zhao, 2020). Indeed, since hyper-reactivity is one of the determinants of COVID-19 critical phase, Everolimus could be utilized for the same rational use as Tocilizumab, Hydrochloroquine, Heparin, and Steroids in the intensive therapy of COVID-19 (Askanase et al., 2020; Chen et al., 2020; Geng et al., 2020; Li et al., 2020; Nikolich-Zugich et al., 2020; Piva et al., 2020; Qin et al., 2020; Radbel et al., 2020; Whyte et al., 2020; Ye et al., 2020; Yuki et al., 2020; Zhao, 2020). Moreover, the putative anti-replicative effect of Everolimus in controlling viral spread could also be promising in SARS-CoV2 infection (**Figure 1**) on the basis of its ability to reduce mRNA translation, ribosome biogenesis, protein synthesis, mitochondrial metabolism, and viral replication (Dunlop and Tee, 2009; Laplante and Sabatini, 2009; Dowling et al., 2010; Garofalo et al., 2019; Nanmoku et al., 2019; Tan et al., 2019).

Honestly, the authors of this short opinion do not have an answer; they aim only to propose to clinicians the hypothesis of modulating the immune response by acting on mTor, as a main immune-regulating key molecule, in the complex disease of SARS-CoV2 infection.

## Author Contributions

GR and GT contributed equally, conceptualized the paper, and wrote the manuscript. VR, AP, AG, and FC contributed to the manuscript and read, edited, and approved the submitted version.

## Conflict of Interest

The authors declare that the research was conducted in the absence of any commercial or financial relationships that could be construed as a potential conflict of interest.
